# Mouse strain-dependent neutralizing antibody responses to Zika virus vaccines

**DOI:** 10.71150/jm.2504005

**Published:** 2025-08-31

**Authors:** Sang Hwan Seo, Jung-ah Choi, Eunji Yang, Hayan Park, Dae-Im Jung, Jae-Ouk Kim, Jae Seung Yang, Manki Song

**Affiliations:** Science Unit, International Vaccine Institute, Seoul 08826, Republic of Korea

**Keywords:** zika virus, neutralizing antibody, vaccine, mouse strain, C57BL/6, BALB/C

## Abstract

The 2015 Zika virus (ZIKV) outbreak in Brazil and its global spread underscored the urgent need for effective and broadly protective vaccines. While C57BL/6 and BALB/c mice are widely used in preclinical vaccine research, direct comparisons of their ability to elicit ZIKV-specific neutralizing antibodies (nAbs) remain limited. This study aimed to systematically evaluate and compare the immunogenic potential of these two common mouse strains across diverse vaccine platforms, focusing on their capacity to generate functional neutralizing antibody responses. We assessed nAb and IgG responses following four vaccination strategies: (1) DNA vaccine encoding prMEΔTM followed by E protein domain III boost, (2) recombinant EΔTM protein expressed using baculovirus system, (3) formalin-inactivated ZIKV, and (4) live ZIKV. Although both strains generated detectable ZIKV- and E protein-specific IgG, the magnitude and quality of responses varied by vaccine platform and strain. Notably, C57BL/6 mice consistently mounted significantly higher nAb titers than BALB/c mice across all immunization groups, including subunit- and whole-virus-based vaccines. In contrast, BALB/c mice showed lower or undetectable nAb responses, despite comparable or higher total IgG levels in some cases. These findings show that host genetic background is a critical determinant of vaccine-induced neutralization and underscore the importance of selecting appropriate animal models in ZIKV vaccine development. C57BL/6 mice, due to their robust nAb responses, represent a reliable model for evaluating vaccine immunogenicity. Conversely, the limited nAb responses in BALB/c mice position them as a potential low-responder model, offering a stringent system to test the potency and breadth of protective immunity under suboptimal conditions.

## Introduction

Emerging and re-emerging viruses have been continuously threatening the health of populations globally. One of the representative viruses is the Zika virus (ZIKV). ZIKV was first isolated from a sentinel rhesus monkey in the Zika forest of Uganda in 1947 (Dick et al., 1952). Since then, three outbreaks of the virus have been witnessed, which are as follows: Asian genotype ZIKV in Yap Island and Guan, Micronesia in 2007, in French Polynesia between 2013 and 2014, and in Brazil in 2015 (Duffy et al., 2009; Fauci and Morens, 2016; Lazear and Diamond, 2016). The last outbreak in Brazil caused more than 30,000 cases of infection which were associated with fetal and newborn microcephaly, and neurological complications in adults such as Guillain-Barre syndrome (Faria et al., 2016; Krauer et al., 2017). The virus belongs to the family Flaviviridae and its genome comprises a single stranded, positive-sense RNA about 10 kb in length encoding three structural proteins, which include the capsid (c), pre-membrane (prM) and envelope (E), and seven non-structural (NS) proteins like NS1, NS2A, NSB, NS3, NS4A, NS4B, and NS5. Among viral proteins, the E protein binds to the membrane receptor on the host cells (Lin et al., 2018).

Although no ZIKV vaccines are currently licensed for commercial use, several candidates have progressed through clinical development. Phase I clinical trials have been completed for multiple vaccine platforms, including messenger RNA (mRNA), inactivated virus, and DNA-based vaccines (Barrett, 2018; Woodson and Morabito, 2024). An mRNA vaccine encoding the prME region from the 2007 Micronesia ZIKV isolate (mRNA-1325) failed to induce detectable ZIKV-specific neutralizing antibodies (nAbs) in 75 volunteers. In contrast, another mRNA vaccine encoding prME from the RIO-U1 isolate achieved 100% seroconversion, with Flavivirus-seronegative participants seroconverting after two doses and Flavivirus-seropositive individuals after a single dose (Essink et al., 2023). Formalin-inactivated ZIKV vaccines have shown variable immunogenicity depending on prior Flavivirus exposure. One candidate induced 100% seroconversion in Flavivirus-naïve participants (n = 122), but less than 80% in those with pre-existing Flavivirus immunity (Han et al., 2021). In a separate trial, another formalin-inactivated vaccine induced seroconversion in 95% of recipients (52 of 55 recipients), and passive transfer of purified IgG from vaccinated individuals conferred protection against viremia in mice (Modjarrad et al., 2018). DNA vaccine candidates encoding prM and E have also been tested. One electroporation-delivered DNA vaccine elicited nAbs in 62% of participants (24 of 39 participants), with titers ranging from 18 to 317 (Tebas et al., 2017). Moreover, sera from these individuals conferred protection against ZIKV in murine challenge models. A needle-free DNA vaccine encoding the same antigens induced nAbs in all recipients (14 of 14 recipients) after a three-dose regimen, with a mean titer of 304 (Gaudinski et al., 2018). While these studies demonstrate the potential of various ZIKV vaccine platforms to induce protective nAbs, immunogenicity varied widely across individuals, and a subset of participants failed to achieve seroconversion. These findings highlight the need for further optimization of vaccine design and dosing strategies, as well as the importance of identifying host factors influencing vaccine responsiveness.

To develop more effective ZIKV vaccine and vaccination strategy, various vaccine candidates should be assessed in terms of ZIKV-specific nAb induction in pre-clinical study and in clinical study. Vaccine-induced nAb titer can be vary by mouse strain used in pre-clinical study (Han et al., 2012; Hocart et al., 1989; Li et al., 2015; Milich et al., 1995, 1997). In a pre-clinical study for ZIKV vaccine candidate using DNA expressing the prM and E proteins of ZIKV, the vaccine-induced ZIKV-specific nAb was more induced in C57BL/6 mice compared to BALB/C mice (Dowd et al., 2016). However, it has not been defined whether the different ZIKV-specific nAb induction by mouse strain is general feature of ZIKV antigen (Ag). Unveiling relationship between mouse strain and ZIKV-specific nAb induction can provide a basis to select proper mouse model for assessment of ZIKV vaccine candidate as well as immunological characteristic of ZIKV Ag.

In this study, we systematically compared ZIKV-specific neutralizing antibody (nAb) responses in BALB/c and C57BL/6 mice following four distinct immunization strategies: (1) a DNA vaccine encoding prME∆TM followed by boosting with recombinant E protein domain III, (2) recombinant E∆TM protein expressed using baculovirus system, (3) formalin-inactivated ZIKV, and (4) live ZIKV. Across all vaccine platforms, C57BL/6 mice consistently elicited significantly higher ZIKV-specific nAb titers than BALB/c mice. These findings indicate that mouse genetic background plays a critical role in shaping the magnitude of vaccine-induced neutralizing responses. Importantly, our results underscore the necessity of carefully selecting preclinical models in vaccine development, as reliance on a single mouse strain may lead to underestimation or overestimation of immunogenicity. Furthermore, the consistently low nAb response observed in BALB/c mice suggests their potential utility as a “low-responder” model for evaluating the robustness of candidate ZIKV vaccines under suboptimal immunological conditions.

## Materials and Methods

### Ethics approval

All animal experiments were approved by the Animal Experiment Committee of International Vaccine Institute with the approval number (IACUC 2016-008).

### Virus, cells, and bacteria

The ZIKV strain fortaleza/2015 (Brazil) was procured from the Walter Reed Army Institute of Research (WRAIR), propagated in Vero cells with a multiplicity of infection (MOI) of ~ 0.1–1, and harvested 3–4 d after the time of initial infection. Supernatants were centrifuged (5,000 rpm, 4°C, 30 min) to remove debris, aliquoted, and stored at -80°C. Plaque assays confirmed consistent viral titers. Vero cells (ATCC CCL-81) were cultured in ATCC-formulated Eagle’s minimum essential medium (EMEM) (ATCC, USA) with 10% heat-inactivated FBS (Invitrogen, USA) and 1% penicillin/streptomycin (Gibco, 100 units/ml and 100 μg/ml) at 37°C in 5% CO₂. *Spodoptera frugiperda* 9 (Sf9) insect cells were used to express E∆TM protein via a baculovirus system and maintained at 27°C in serum-free medium (Thermo Fisher Scientific, USA). For expression of ZIKV E protein, coating antigen used in ELISA, *E. coli* BL21 cells cultured at 37°C in LB broth (Scharlau, Spain) supplemented with 100 μg/ml ampicillin (Sigma-Aldrich, Cat# A5345, USA).

### Preparation of ZIKV vaccines

A DNA vaccine encoding prME∆TM was designed based on the ZIKV Brazil strain SPH2015 (GenBank: KU321639.1), with codon optimization for mammalian expression and inclusion of a Japanese encephalitis virus signal sequence (Dowd et al., 2016). The gene was synthesized (Cosmogenetech, Korea) and cloned into the pcDNA3.1(–) vector (Thermo Fisher Scientific, USA) using EcoRI and BamHI sites. Commercial E protein domain III was purchased from Alpha Diagnostics (cat# ZENV17-R-100, USA). For preparation of baculovirus expressing E∆TM, DNA plasmid with 6× His-tag was synthesized as described above and cloned in a pFastBac vector carrying a signal peptide (gp67) of the pAcGP67 vector (provided by Dr. Hyun-Soo Jo, Yonsei Univ.) using the 5´- and 3´- restriction enzyme sites of BamHI and XhoI, respectively. The culture supernatant of Sf9 cells, three days post transfection of the plasmid DNA, was harvested; this was the first passage (P1) of the recombinant baculovirus. The Sf9 cells were infected with a P3 preparation of baculoviruses at a multiplicity of infection (MOI) of 5 for purification of the E∆TM protein. The infected cells were incubated at 27℃ under conditions of shaking for 4 days, after which, the cell culture media was centrifuged, the supernatant was concentrated using the SARTOFLOW^®^ Slice 200 Benchtop Crossflow System (Sartorius AG, Germany) and purified using the ÄKTA start affinity chromatography (GE Healthcare, Sweden). The HisTrap resin was equalized using urea buffer and the sample supernatant was loaded onto the resin for binding. After washing twice with 100 ml each of binding buffer and 35 mM binding buffer (binding buffer + imidazole 35 mM) sequentially, 500 mM imidazole buffer (binding buffer + imidazole 500 mM) was loaded onto the resin to collect the fraction (total 2 ml/tube, 30 tubes). The imidazole in the fraction was removed using an Amicon tube (Millipore, USA) and the protein concentration and purity were measured using the BCA kit (Thermo Fisher Scientific, USA) and SDS-PAGE, respectively. For preparation of the formalin-inactivated ZIKV vaccine, live ZIKV was purified using Capto Core 700 (GE Healthcare, USA). The purified ZIKV was inactivated by incubation with 0.05% formalin for 48 h at 22°C.

### Expression of ZIKV E protein using *E. coli*

The ZIKV E protein was expressed in *E. coli* for use as a coating antigen in ELISA. The gene encoding the E protein was synthesized (Cosmogenetech, Korea) and cloned into the pET28a vector using NcoI and XhoI restriction sites, then transformed into *E. coli* BL21 (Lucijen, USA). For induction of the E protein expression in *E. coli*, the bacteria were incubated for 4 h with 0.5 M isopropyl β-D-1-thiogalactopyranoside and the cultured pellets were suspended using a binding buffer (20 mM tris, 500 mM NaCl, 10% glycerol, and pH 7.9) followed by sonication and centrifugation to collect the pellet. The pellets were resuspended in urea buffer (binding buffer containing 6 M urea, pH 7.9) and incubated overnight at 4℃. After centrifugation, the supernatant was collected and purified by affinity chromatography as described previously.

### Western blot

To confirm the expression of ZIKV E protein from plasmid DNA, HEK293T cells (3 × 10⁵) were seeded in 6-well plates. The next day, the cells were transfected with 2.5 µg of pcDNA-encoding prME or prME∆TM using Lipofectamine 2000. After 48 h, cells were lysed with RIPA buffer (SIGMA, USA), and lysates were subjected to SDS-PAGE on a 10% gel (Bio-Rad, USA), followed by transfer to a polyvinylidene difluoride (PVDF) membrane (Bio-Rad, USA). The membrane was blocked with 10% non-fat milk in tris-buffered saline (TBS) (Bio-Rad, USA) for 4 h, then incubated overnight at 4°C with anti-ZIKV E protein domain III mAb (1:500, Alpha Diagnostic, cat# ZEND20-A). After washing, the membranes were incubated with HRP-conjugated goat anti-mouse IgG (1:10,000, Abcam, cat# ab6721), washed again, and developed using ECL substrate. Commercial ZIKV E protein expressed in Sf 9 cells (Alpha Diagnostic, Cat# ZENV16-R-10) was used as positive control.

Expression of the E∆TM protein and prM in Sf9 cells infected with recombinant baculovirus was confirmed by Western blot analysis. Sf9 cells were seeded at a density of 2 × 10⁶ cells/ml and infected with P3-stage recombinant baculovirus encoding E∆TM or prME∆TM at a MOI of 5. The infected cells were cultured in a shaking incubator at 27°C for 4 days. Following incubation, both cell pellets and culture supernatants were harvested and processed for Western blotting as described above. Membranes were probed with a monoclonal anti-Zika virus envelope domain III antibody (1:500, Alpha Diagnostic, Cat# ZEND20-A) or a polyclonal anti-ZIKV prM antibody (1:1,000, GeneTex, Cat# GTX133305), followed by horseradish peroxidase (HRP)-conjugated goat anti-rabbit IgG secondary antibody (1:10,000, Abcam, Cat# ab6721).

### Mice immunization

Five-week-old female BALB/c and C57BL/6 mice (Koatech, Korea) were maintained under specific pathogen-free (SPF) conditions and acclimated for one week prior to immunization. Mice were intramuscularly immunized in the quadriceps muscle three times at two-week intervals. Immunization groups included mice receiving PBS as a negative control, a DNA vaccine encoding prME∆TM (50 μg per dose, administered twice) followed by a single protein boost with 1 μg of recombinant E protein domain III expressed in HEK-293 cells, or baculovirus-expressed E∆TM protein administered at 1, 5, or 10 μg per dose. Additional groups received three doses of either formalin-inactivated ZIKV (10 μg per dose) or live ZIKV (10 μg per dose), with the latter serving as a positive control. All vaccines were administered without adjuvant in a total volume of 50 μl per injection. The immunization doses were selected based on previous studies, including 10 μg for formalin-inactivated ZIKV and 50 μg for DNA vaccines (Larocca et al., 2016; Sumathy et al., 2017). For the ZIKV E protein domain III, previous reports have used doses ranging from 10 to 25 μg in mice, depending on protein source, formulation, and use of adjuvants (Cibulski et al., 2021; Lin et al., 2019; Yang et al., 2017). In our study, the dose of ZIKV E protein domain III was reduced to 1 μg in consideration of the two prior DNA priming doses, which were expected to elicit antigen-specific memory responses and enhance the effect of the protein boost. The highest dose of recombinant E∆TM protein (10 μg) and the dose of live virus were selected to match the total antigen content used in the formalin-inactivated ZIKV group, allowing for consistent comparisons across vaccine platforms. Blood samples were collected via submandibular bleeding on days 13, 27, and 41 after the initial immunization. Sera were used to quantify ZIKV-specific IgG titers, as well as E protein-specific IgG, IgG1, and IgG2a isotypes by ELISA. Neutralizing antibody titers were determined using a plaque reduction neutralization test (PRNT).

### Enzyme-linked immunosorbent assay

To determine the titers of ZIKV- or E protein-specific Abs, 96-well plates (Thermo Fisher Scientific, USA) were coated with 6 µg/ml of formalin-inactivated ZIKV (Brazil strain) or 2 µg/ml of ZIKA E protein expressed using *E. coli* in PBS for 16 h at 4℃, respectively. The coated plates were washed thrice with PBS (Thermo Fisher Scientific, USA) containing 0.05% Tween 20 (PBST), incubated with PBS in 1% BSA (Merck, Germany, blocking buffer) for 1 h at 37℃, and washed thrice with PBST. For the antibody reaction, the serum samples from vaccinated mice were diluted to 1:30 using the blocking buffer, and 5-fold serially diluted samples were added to the plates followed by incubation for 1 h at 37℃. After washing with PBST thrice, 1:3,000 diluted goat anti-mouse IgG (cat# 1030-05, HRP-conjugated, Southern Biotech, USA), IgG1 (cat# 1070-05, HRP-conjugated, Southern Biotech) or IgG2a antibodies (cat# 1080-05, HRP-conjugated, Southern Biotech) in blocking buffer containing 0.05% Tween 20 were added to each well and incubated for 1 h at 37℃. It was washed again with PBST thrice and TMB solution (Millipore, USA) was added to each well followed by incubated at RT. The color development was ceased by adding 0.5 M HCl (Merck, Germany) and the optical density (OD) values were measured at 450 nm wavelength using an ELISA reader (Molecular Devices, USA). The OD cut-off value of 0.2 was determined as the endpoint titer.

### Plaque reduction neutralization test (PRNT)

The plaque reduction neutralization test (PRNT) was performed with slight modifications from a previous method (Sapparapu et al., 2016). Diluted sera were incubated with ZIKV for 1 h at 37°C and added to Vero cells, followed by 30 min incubation. The cells were then overlaid with 0.8% low-melting agarose in EMEM containing 2% heat-inactivated FBS. After 3–4 days, the plates were fixed with 4% formaldehyde (Sigma, USA) and stained with 1% crystal violet (Sigma, USA) for 1 h. The stained plates were rinsed with tap water and allowed to air-dry at room temperature. The nAb titer was determined as the serum dilution at which 50% of the virus inoculum was inhibited.

### Statistical analysis

Statistical analysis was performed using one-way ANOVA with GraphPad Prism (GraphPad Software, USA). Differences between two groups were assessed using two-tailed Student’s t-tests. P-values < 0.05 were considered statistically significant.

## Results

### Expression and secretion of ZIKV prME∆TM and E∆TM proteins in baculovirus and DNA vaccine systems

To compare ZIKV- and E protein-specific binding antibody (Ab) titers as well as ZIKV-specific neutralizing antibody (nAb) responses in BALB/c and C57BL/6 mice, we prepared a panel of ZIKV vaccine candidates, including: (i) prME∆TM protein expressed using the baculovirus expression system, (ii) a DNA vaccine encoding prME∆TM, (iii) formalin-inactivated ZIKV, and (iv) live ZIKV.

**Fig. 1.**
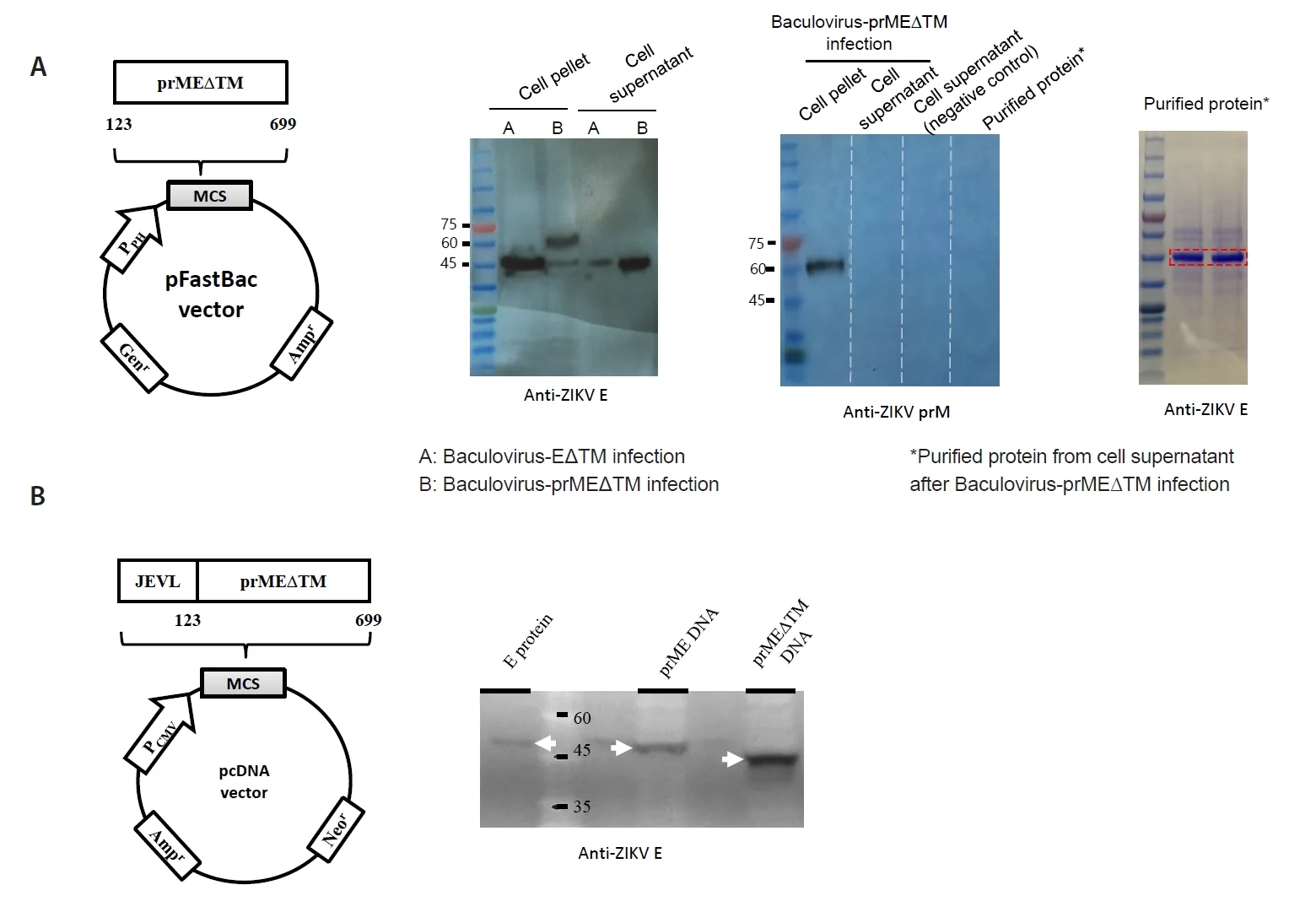
Expression of ZIKV E and prME∆TM proteins from baculovirus and DNA constructs. (A) Western blot analysis of cell pellets and culture supernatants from Sf9 cells infected with recombinant baculoviruses expressing either prME∆TM or E∆TM. Samples were probed with anti-ZIKV E and anti-ZIKV prM monoclonal antibodies. (B) Western blot analysis of HEK-293 cells transfected with pcDNA constructs encoding either prME or prME∆TM. Cell pellets and supernatants were probed with an anti-ZIKV E monoclonal antibody. A commercially available ZIKV E protein expressed in Sf9 cells was included as a positive control for Western blot analysis. Amp^r^: ampicillin-resistant gene, JEVL: Japanese encephalitic virus leader sequence, MCS: multi-cloning site, Neo^r^: neomycin resistant gens, P_CMV_: promoter of cytomegalovirus.

To confirm antigen expression from the baculovirus-prME∆TM construct, Western blot analysis was performed using anti-ZIKV E and anti-ZIKV prM antibodies on cell pellets and culture supernatants collected after infection (Fig. 1A). In both the cell pellet and the supernatant following infection with baculovirus-E∆TM (used as a comparator virus), a ~45 kDa band corresponding to the expected size of E∆TM was observed. In contrast, the cell pellet from baculovirus-prME∆TM-infected cells displayed two bands: one at ~64 kDa (consistent with prME∆TM) and another at ~45 kDa (E∆TM). Notably, only the ~45 kDa band was detected in the culture supernatant, indicating that while both prME∆TM and E∆TM proteins are present intracellularly, only E∆TM is secreted. To further validate this observation, Western blot analysis using an anti-ZIKV prM antibody revealed that the ~64 kDa prME∆TM band was exclusively present in the cell pellet, not in the supernatant. These findings suggest that cleavage of prM from prME∆TM results in the release of E∆TM into the extracellular space. Consistent with this, the purified protein from the supernatant of baculovirus-prME∆TM-infected cells showed only the ~45 kDa E∆TM band.

To assess protein expression from the DNA vaccine construct, HEK-293 cells were transfected with pcDNA-prME∆TM, and Western blot analysis was performed on both cell pellets and supernatants (Fig. 1B). The supernatant from pcDNA-prME-transfected cells showed a band corresponding in size to the E protein, similar to the baculovirus system, indicating that the full-length prME protein is retained intracellularly, while only the cleaved E protein is secreted. In contrast, transfection with pcDNA-prME∆TM resulted in a smaller E protein band in the supernatant, consistent with deletion of the transmembrane domain. Taken together, these results demonstrate that both baculovirus- and DNA-based prME∆TM constructs efficiently express and secrete the truncated ZIKV E protein, supporting their use as vaccine antigens.

### Mouse strain-dependent ZIKV-specific IgG responses elicited by diverse vaccine platforms

**Fig. 2.**
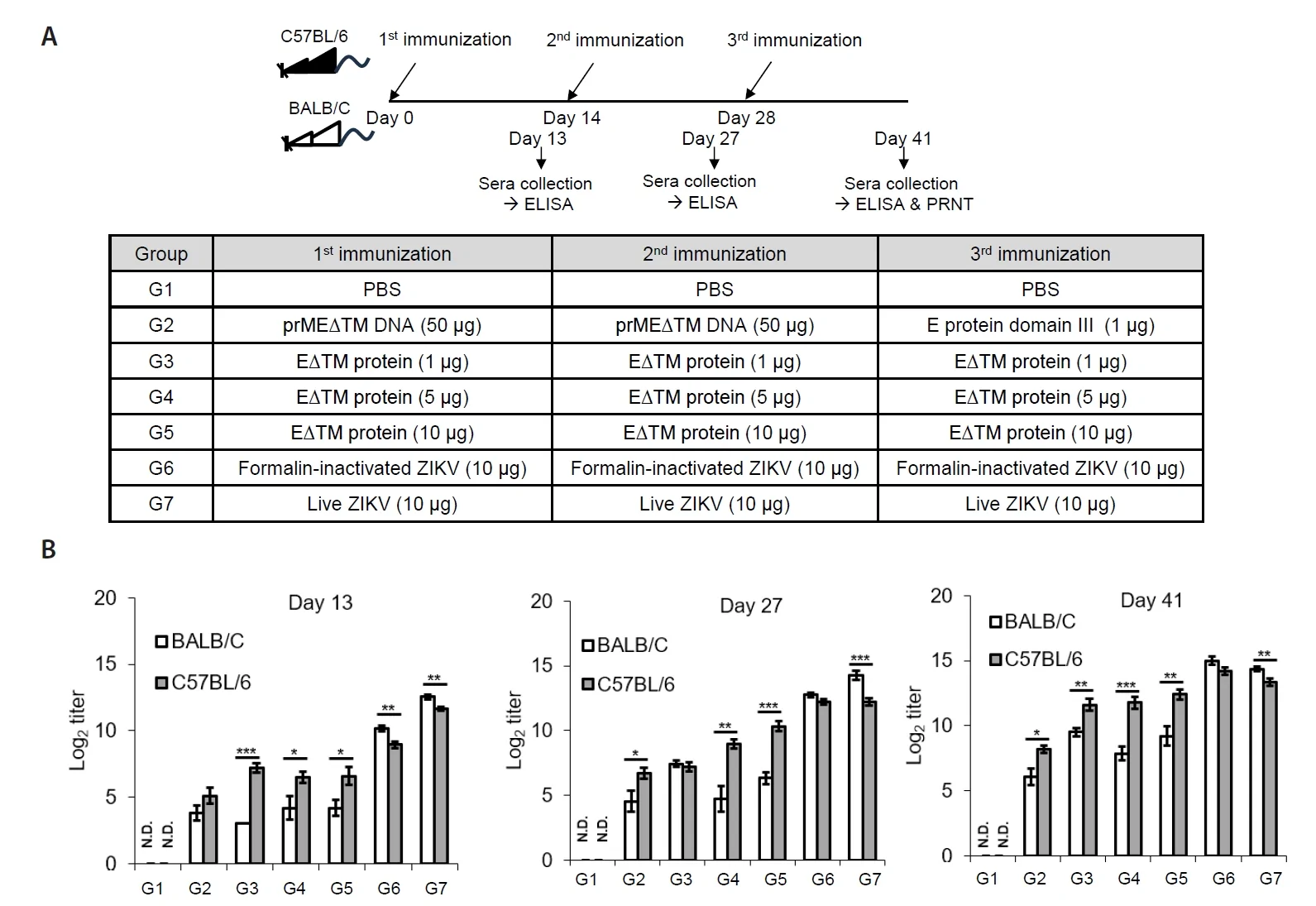
Differences in ZIKV-specific IgG titers induced by various ZIKV vaccine candidates in C57BL/6 and BALB/c mice. (A) Schematic diagram of the immunization schedule used to evaluate the immunogenicity of each vaccine in C57BL/6 and BALB/c mice. Mice (n = 10 per group) were immunized three times as follows: G1, PBS only (negative control); G2, two doses of pcDNA-prME∆TM (50 μg/dose) followed by a single dose of E protein domain III (1 μg) expressed in HEK-293 cells; G3–G5, three doses of baculovirus-expressed E∆TM protein at 1, 5, or 10 μg per dose; G6, three doses of formalin-inactivated ZIKV (10 μg/dose); G7, three doses of live ZIKV (10 μg/dose). Sera were collected on days 13, 27, and 41 post-initial immunization for assessment of binding antibody titers via ELISA. (B) ZIKV-specific IgG titers measured in C57BL/6 and BALB/c mice across the immunization groups. Data represents the mean ± SEM. Statistical significance among groups was determined using one-way ANOVA (^*^p < 0.05, ^**^p < 0.01, ^***^p < 0.001). ∆TM, transmembrane domain deleted; N.D., not detected.

To assess the immunogenicity of different ZIKV vaccine platforms and compare host strain-specific responses, C57BL/6 and BALB/c mice (n = 10 per group) were immunized with one of seven vaccine regimens (Fig. 2A). Group 1 (G1) received PBS as a negative control. Group 2 (G2) was immunized with two doses of a DNA vaccine encoding prME∆TM (50 μg), followed by a single boost with 1 μg of E protein domain III expressed in HEK-293 cells. Groups 3–5 (G3–G5) received three doses of baculovirus-expressed E∆TM protein at 1, 5, or 10 μg, respectively. Group 6 (G6) received formalin-inactivated ZIKV (10 μg), and Group 7 (G7) was immunized with live ZIKV (10 μg) as a positive control. ZIKV-specific IgG titers were measured by ELISA in serum samples collected on days 13, 27, and 41 post-initial immunization (Fig. 2B).

On day 13, significant IgG responses were observed in groups immunized with E∆TM protein (G3–G5), with C57BL/6 mice exhibiting significantly higher titers than BALB/c mice. In contrast, IgG titers were higher in BALB/c mice following immunization with formalin-inactivated (G6) and live ZIKV (G7). By day 27, antibody titers increased markedly in both strains. C57BL/6 mice showed significantly higher IgG levels in all groups except immunized with 1ug of E∆TM protein or formalin-inactivated ZIKV compared to BALB/c mice. These trends persisted on day 41, where C57BL/6 mice exhibited higher titers in all groups except mice immunized with formalin-inactivated ZIKV, where responses were comparable between strains. The highest titers were observed in the live ZIKV group (G7) in both strains, with no significant difference, suggesting strong immunogenicity regardless of host background. Overall, these results demonstrate that while all vaccine candidates elicited robust ZIKV-specific IgG responses, C57BL/6 mice generally mounted stronger humoral responses than BALB/c mice, highlighting the impact of host genetic background on vaccine-induced immunity.

### ZIKV E protein-specific IgG titers vary by vaccine type and mouse strain

**Fig. 3.**
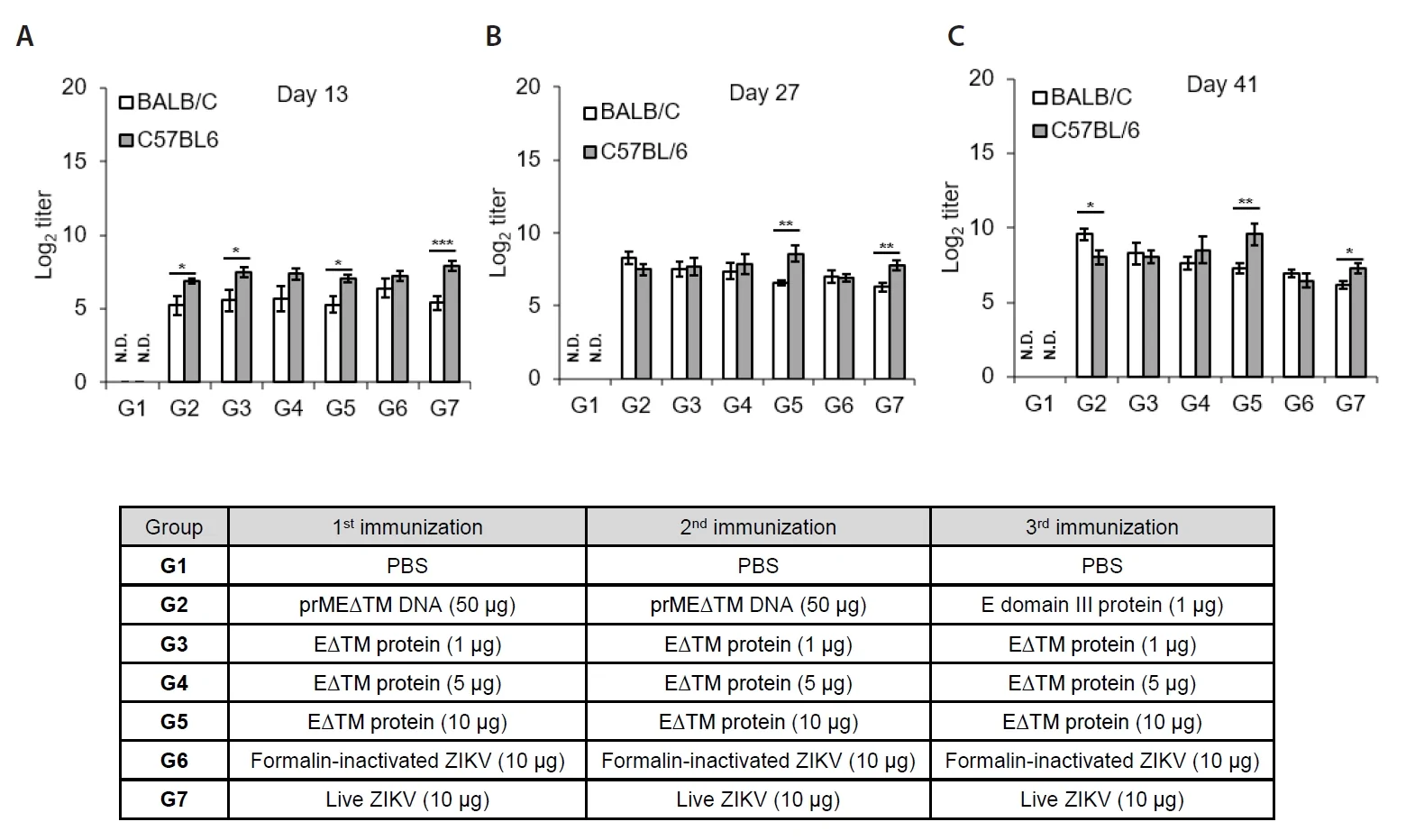
ZIKV E protein-specific IgG responses in C57BL/6 and BALB/c mice following immunization with various ZIKV vaccine platforms. Serum samples collected on days 13 (A), 27 (B), and 41 (C) post-initial immunization were analyzed for ZIKV E protein-specific IgG titers by ELISA using recombinant E protein expressed in *E. coli* as the coating antigen. Mice were immunized with PBS (G1); prME∆TM DNA (50 μg, administered twice) followed by a single dose of E protein domain III (1 μg) (G2); baculovirus-expressed E∆TM protein at doses of 1, 5, or 10 μg (G3–G5); formalin-inactivated ZIKV (10 μg, G6); or live ZIKV (10 μg, G7), according to the immunization schedule shown in Fig. 2. IgG titers are presented as mean ± SEM (n = 10 per group). Statistical significance between strains was determined by one-way ANOVA (^*^p < 0.05, ^**^p < 0.01, ^***^p < 0.001). N.D., not detected. (Bottom) Immunization schedule table summarizing the formulations and dosages administered at each time point (1st, 2nd, and 3rd immunizations) for each experimental group.

While total IgG responses reflect overall humoral reactivity to multiple viral antigens present in the virion, E protein-specific responses more directly evaluate antibodies targeting the major surface antigen responsible for virus entry and neutralization. Thus, we assessed the levels of E protein-specific IgG in C57BL/6 and BALB/c mice following immunization with various ZIKV vaccine candidates (Fig. 3). At day 13 (Fig. 3A), all vaccine groups except PBS (G1) elicited detectable ZIKV E protein-specific IgG responses. C57BL/6 mice consistently showed higher titers than BALB/c mice in groups receiving DNA + E protein domain III (G2), E∆TM protein (1 µg [G3] and 10 µg [G5]), with the greatest difference observed in the live ZIKV group (G7, *p* < 0.001). By day 27 (Fig. 3B), IgG titers increased in both strains. C57BL/6 mice continued to exhibit higher responses than BALB/c mice, particularly in the 10 μg E∆TM group (G5) and live ZIKV group (G7), both showing statistically significant differences (*p* < 0.01). On day 41 (Fig. 3C), seven days after the final immunization, the IgG titers varied by group and strain. Notably, BALB/c mice immunized with the DNA + E protein domain III regimen (G2) showed higher titers than C57BL/6 mice (*p* < 0.05), suggesting a delayed but strong response. However, C57BL/6 mice maintained significantly higher IgG titers in the 10 μg E∆TM (G5) and live ZIKV (G7) groups compared to BALB/c mice. In contrast, the 1 μg and 5 μg E∆TM (G3, G4) and formalin-inactivated ZIKV (G6) groups showed no statistically significant differences between strains. The highest overall titers were observed in C57BL/6 mice receiving 10 μg E∆TM (G5), while the lowest titers were seen in BALB/c mice immunized with live ZIKV (G7). These results demonstrate that ZIKV E protein-specific IgG responses vary by both vaccine platform and host genetic background. C57BL/6 mice generally mounted more robust early responses, particularly to protein- and live virus-based vaccines, while BALB/c mice showed relatively weaker and sometimes delayed responses, especially in the live virus group.

### Mouse strain and vaccine platform influence the balance of IgG1 and IgG2a responses

**Fig. 4.**
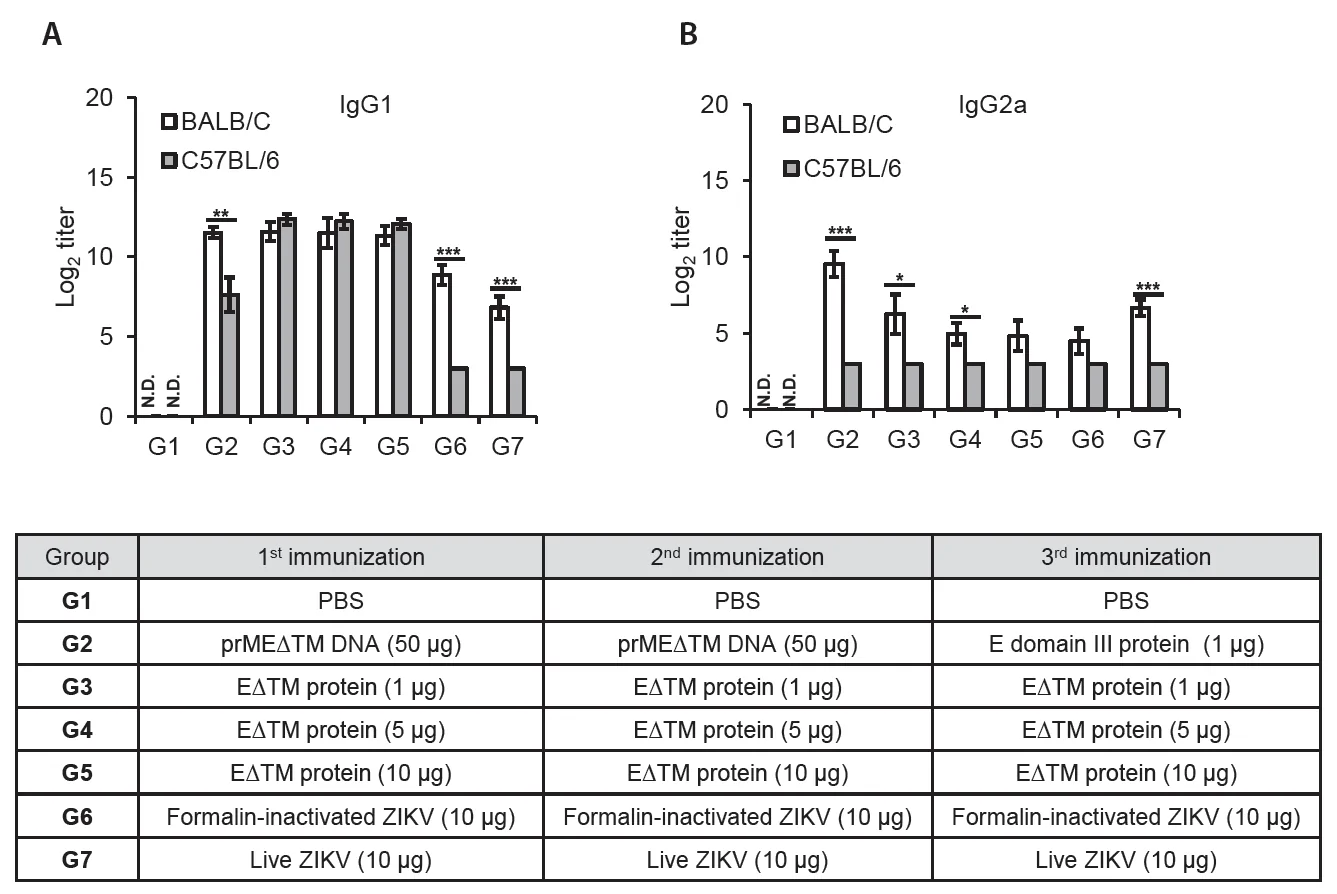
ZIKV E protein-specific IgG1 and IgG2a responses in C57BL/6 and BALB/c mice following immunization with various ZIKV vaccine platforms. Serum samples collected on day 41 post-initial immunization were analyzed by ELISA to quantify IgG1 (A) and IgG2a (B) isotype titers specific to ZIKV E protein. Recombinant E protein expressed in *E. coli* was used as the coating antigen. Mice were immunized with various ZIKV vaccine candidates according to the schedule outlined in Fig. 2, including PBS (G1), prME∆TM DNA (50 μg, twice) followed by E protein domain III (1 μg) (G2), baculovirus-expressed E∆TM protein at three dose levels (G3–G5), formalin-inactivated ZIKV (G6), or live ZIKV (G7). Antibody titers are presented as mean ± SEM (n = 10 per group). Statistical comparisons between mouse strains were performed using one-way ANOVA (^*^p < 0.05, ^**^p < 0.01, ^***^p < 0.001). N.D., not detected. The table presents the vaccine formulations and corresponding doses used for each immunization group.

To investigate the qualitative nature of the humoral response induced by different ZIKV vaccine platforms, we measured ZIKV E protein-specific IgG1 and IgG2a titers in sera from BALB/c and C57BL/6 mice collected on day 41 post-initial immunization (Fig. 4). As shown in Fig. 4A, all vaccine groups (except PBS control) induced robust IgG1 responses, indicative of Th2-skewed immunity. Notably, BALB/c mice showed significantly higher IgG1 titers than C57BL/6 mice in the formalin-inactivated ZIKV (G6) and live ZIKV (G7) groups (*p* < 0.001), while the DNA + E protein domain III group (G2) also showed a modest but significant increase in BALB/c mice (*p* < 0.01). IgG1 responses were comparable between strains in protein-only groups (G3–G5). The IgG2a isotype—often associated with Th1-type responses—was markedly higher in BALB/C mice across nearly all vaccine groups (Fig. 4B). Particularly, the DNA + E protein domain III group (G2) induced strong IgG2a titers in BALB/C mice, significantly exceeding those observed in C57BL/6 mice (*p* < 0.001). Similar trends were observed in protein-based groups (G3, G4) and the live virus group (G7), with BALB/C mice displaying higher IgG2a titers, while IgG2a titers in C57BL/6 mice remained low across all groups. These findings demonstrate that antibody isotype responses vary by mouse strain and vaccine platform. C57BL/6 mice showed strong IgG1 but low IgG2a responses, while BALB/c mice produced high levels of both isotypes, indicating that Th1/Th2 polarization is influenced by both host genetics and vaccine type.

### C57BL/6 mice exhibit stronger neutralizing antibody responses to ZIKV vaccines than BALB/c mice

**Fig. 5.**
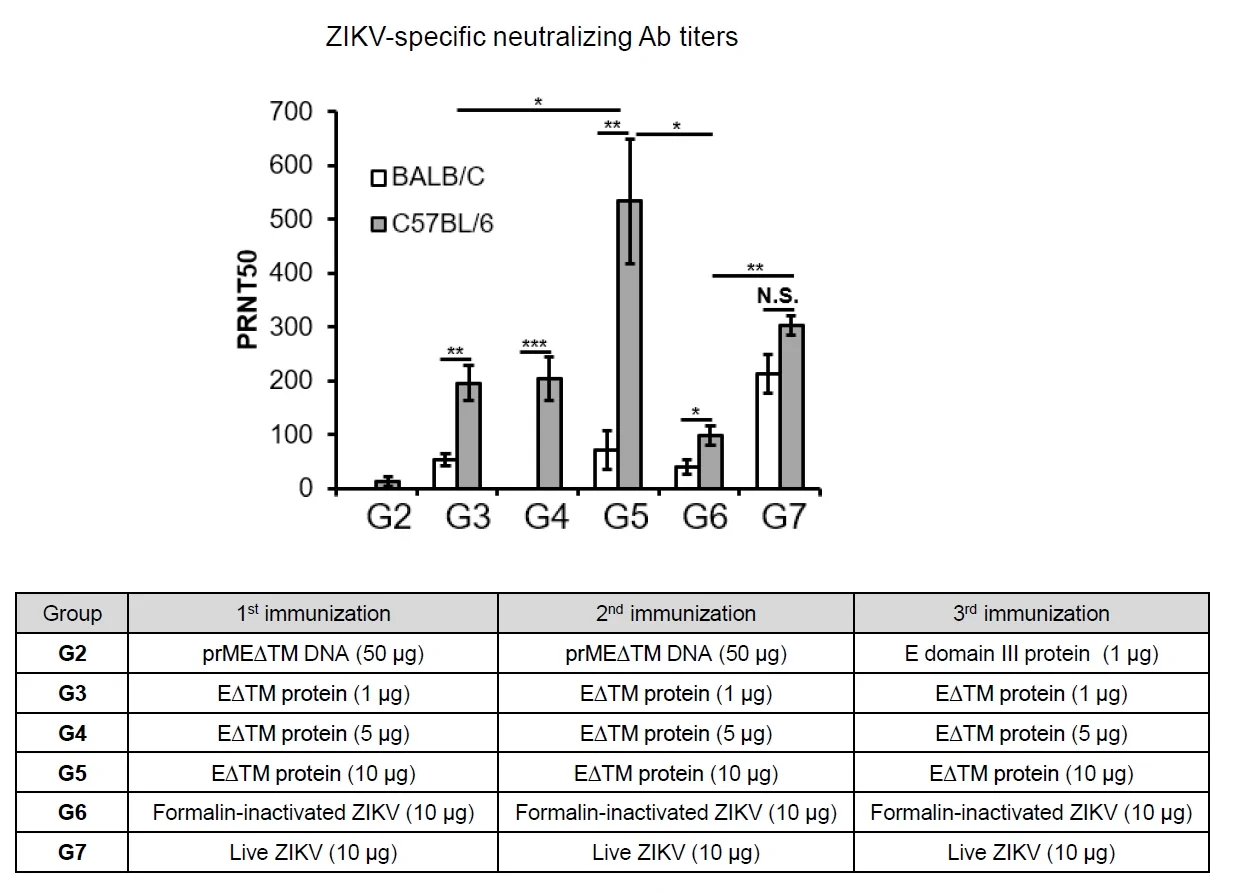
Neutralizing antibody responses induced by various ZIKV vaccines in C57BL/6 and BALB/c mice. ZIKV-specific neutralizing antibody titers were measured by plaque reduction neutralization test (PRNT) on day 41 post-initial immunization. PRNT50 values represent the serum dilution at which a 50% reduction in ZIKV plaque formation was observed. Mice were immunized with prME∆TM DNA (50 μg, twice) followed by E protein domain III (1 μg, once) (G2), baculovirus-expressed E∆TM protein at 1, 5, or 10 μg (G3–G5), or formalin-inactivated ZIKV (10 μg, G6), as described in the immunization schedule in Fig. 2. Data are shown as mean ± SEM (n = 10 per group). Statistical comparisons were performed between groups and between strains using one-way ANOVA (^*^p < 0.05, ^**^p < 0.01, ^***^p < 0.001, N.S., not significant). The table summarizes the vaccine formulations and dosing schedules for each group.

To evaluate the functional capacity of vaccine-induced antibodies, ZIKV-specific neutralizing antibody titers were assessed by plaque reduction neutralization test (PRNT) on day 41 post-initial immunization. As shown in Fig. 5, C57BL/6 mice exhibited significantly higher PRNT50 titers than BALB/c mice in all groups, except live ZIKV immunized group. Among these, the 5 μg E∆TM protein group (G4) induced the highest neutralizing antibody titers in C57BL/6 mice, significantly exceeding all other groups (*p* < 0.05 to *p* < 0.01). In contrast, BALB/c mice showed low or undetectable neutralizing antibody titers in most vaccine groups, except for the live ZIKV group, where robust responses were observed. Notably, no significant difference in PRNT50 titers was found between the two strains in the live virus group, suggesting that this platform elicits comparable neutralizing responses regardless of genetic background. These findings highlight that vaccine-induced neutralizing antibody responses are strongly influenced by host genetic background, with C57BL/6 mice mounting markedly more robust responses than BALB/c mice across most vaccine platforms. The significantly higher PRNT50 titers observed in C57BL/6 mice, particularly following DNA and protein immunization, underscore their heightened capacity to generate functional, virus-neutralizing antibodies. These results suggest that C57BL/6 mice are more responsive for evaluating neutralizing antibody-based immunogenicity of ZIKV vaccine candidates, while BALB/c mice may underestimate the protective potential of certain platforms.

## Discussion

In this study, we found that C57BL/6 mice, across all vaccine platform tested, consistently exhibited consistently higher ZIKV-specific nAb titers compared to BALB/c mice. These findings extend previous observations made with DNA vaccines alone (Dowd et al., 2016), suggesting that enhanced nAb induction in C57BL/6 mice is not platform-specific but instead reflects a broader strain-dependent immunological feature. Although both strains exhibited differences in total and E-specific IgG levels, the disparity in functional nAb titers was more pronounced, underscoring the influence of host genetic background on vaccine-induced protective immunity.

The differential neutralizing antibody responses observed between C57BL/6 and BALB/c mice in this study likely reflect intrinsic genetic and immunological differences between the two strains. C57BL/6 mice, which possess an H-2b MHC haplotype, are known to exhibit a Th1-skewed immune response profile with stronger IFN-γ production and greater IgG2a class switching. In contrast, BALB/c mice, carrying the H-2d haplotype, tend to mount Th2-biased responses characterized by elevated IL-4 and IgG1 production (Mills et al., 2000). These distinctions likely affect T helper cell differentiation and the nature of B cell help, thereby shaping the quality and quantity of vaccine-induced humoral immunity. Beyond Th1/Th2 bias, other genetic factors may contribute to the strain-specific vaccine responses. Differences in pattern recognition receptor (PRR) signaling, such as through TLR7 and TLR9, can modulate innate immune sensing of nucleic acid vaccines and viral components, influencing dendritic cell activation and cytokine secretion (Iwasaki and Medzhitov, 2015; Schenten and Medzhitov, 2011). These early events critically shape the magnitude and polarization of subsequent adaptive responses. Strain-dependent variation in B cell receptor (BCR) repertoire, germinal center dynamics, and affinity maturation efficiency may further influence the development of high-affinity and class-switched antibodies. The expression and function of key regulators such as AID (Aicda), CD40/CD40L, and T follicular helper (Tfh) cell-related factors (e.g., Bcl6, IL-21) are also potential contributors (Crotty, 2011; Wesemann et al., 2013). In addition, polymorphisms in cytokine receptors and their downstream signaling molecules, including STAT6 (IL-4 pathway) and STAT1 (IFN-γ pathway), may influence isotype switching and antibody effector function. Despite these insights, a limitation of our study is that we did not directly identify or validate the specific genetic determinants responsible for the observed strain-dependent responses. Future investigations using genetically engineered mouse models, transcriptomic profiling, or CRISPR-based gene perturbation could help delineate the precise molecular mechanisms by which host genetic background shapes vaccine-induced immunity. Such approaches will be important for translating preclinical findings into predictive models for human vaccine responses, particularly in the context of emerging viral pathogens like ZIKV.

To better characterize the quality and polarization of humoral immune responses, we analyzed the distribution of IgG1 and IgG2a subclasses, which reflect Th2- and Th1-type immune responses, respectively (Snapper and Paul, 1987). While these isotypes provide insight into T helper cell bias, their association with functional protection is context dependent. Interestingly, we found that high IgG1 titers did not consistently correlate with strong neutralizing antibody responses. For example, C57BL/6 mice immunized with the DNA + E protein domain III or high-dose E∆TM protein vaccines developed strong IgG1 responses, yet only the high-dose protein group exhibited robust neutralizing titers. Similarly, BALB/c mice showed elevated levels of both IgG1 and IgG2a in response to several vaccine platforms, including inactivated and live virus vaccines, but their neutralizing titers were comparatively lower or absent in most groups. These results indicate that IgG subclass levels alone do not directly predict neutralizing capacity, and that additional factors—such as antibody affinity, epitope specificity, and B cell selection in germinal centers—likely contribute to functional efficacy. Moreover, the observed divergence between isotype magnitude and neutralization suggests that different vaccine platforms may vary not only in the quantity but also the quality of the antibodies they induce. Antigen conformation, persistence, and the extent of T follicular helper cell engagement may influence the maturation of neutralizing antibodies, which is not fully captured by subclass quantification alone. Together, these findings highlight the need to interpret IgG subclass data in conjunction with functional assays such as PRNT and support the use of multiple complementary readouts to assess vaccine-induced immunity.

Although BALB/c mice generally produced lower levels of ZIKV-specific neutralizing antibodies (nAbs) than C57BL/6 mice following immunization with the DNA vaccine (prMEΔTM), recombinant EΔTM protein, or inactivated ZIKV, this difference was not observed following live ZIKV immunization. This suggests that a defect inherent to the BALB/c immune system may limit nAb production in response to certain vaccine platforms, but that live ZIKV may possess immune-stimulatory components capable of overcoming this limitation. Previous studies have shown that live ZIKV activates the inflammasome pathway via the interaction of its NS5 protein with NLRP3, a pattern recognition receptor containing LRR and PYD domains (Wang et al., 2018). As inflammasome activation is known to enhance antibody production, it is plausible that live ZIKV facilitates a more immunogenic environment in BALB/c mice, thereby enabling nAb induction at levels comparable to those observed in C57BL/6 mice (Eisenbarth et al., 2008; Lee et al., 2018; Li et al., 2008; Sanos et al., 2017; Suschak et al., 2015; To et al., 2018).

Interestingly, inactivated ZIKV elicited significantly lower ZIKV-specific neutralizing antibody (nAb) titers than live ZIKV in both BALB/c and C57BL/6 mice, with the reduction particularly pronounced in BALB/c mice. This diminished response may be attributed to formalin-induced alterations in antigen structure. Formalin inactivation is known to form irreversible methylene bridges between amino acid residues, potentially disrupting conformational epitopes critical for B cell and CD4^+^ T cell recognition (Hassert et al., 2018; Ramos-Vara, 2005). Specifically, modifications to the E protein may hinder its native folding, affecting both the structural integrity of B cell epitopes and the processing of CD4^+^ T cell epitopes. Since CD4^+^ T cell help is essential for the generation of high affinity nAbs, impairment of these epitopes could contribute to the reduced immunogenicity of the inactivated vaccine. Furthermore, formalin inactivation may also impact on internal viral proteins, such as NS5. A recent study demonstrated that infection with live influenza virus induces antibodies against neuraminidase (NA), an internal protein, whereas such responses are absent with inactivated vaccines due to epitope disruption (Chen et al., 2018). Similarly, inactivated ZIKV may impair NS5 structure and its ability to engage in innate immune pathways. Notably, NS5 has been shown to activate inflammasomes via NLRP3 binding (Wang et al., 2018), and such activation may enhance the immunogenic environment required for effective nAb induction. The inability of formalin-inactivated ZIKV to activate this pathway may further compromise its immunostimulatory potential. These findings suggest that both structural and innate immune mechanisms contribute to the superior nAb responses induced by live ZIKV, and that alternative inactivation strategies may be needed to preserve immunogenicity. Further investigation is warranted to clarify the molecular basis of these observations.

Given their consistently robust nAb responses across all vaccine platforms tested, C57BL/6 mice represent a suitable and reliable preclinical model for evaluating the immunogenicity of candidate ZIKV vaccines. In contrast, BALB/c mice exhibited markedly lower ZIKV-specific nAb titers regardless of the vaccine type, indicating an intrinsic limitation in their ability to mount potent neutralizing responses. Consequently, BALB/c mice may serve as a relevant low-responder model for assessing ZIKV vaccine efficacy under immunologically constrained conditions. The concept of responder stratification is well established in the context of hepatitis B virus (HBV) vaccination, where individuals are classified as good responders (anti-HBs ≥ 100 mIU/ml), poor responders (10–100 mIU/ml), and non-responders (< 10 mIU/ml) (Han et al., 2012). Similar patterns have been reported in murine models, with B10.S and C57BL/6 mice representing poor and good responders, respectively, to HBV vaccination (Li et al., 2015; Milich et al., 1995, 1997). These models have provided valuable insights into the immunological mechanisms underlying suboptimal vaccine responses. Analogously, the BALB/c mouse may be used to investigate the determinants of weak nAb induction following ZIKV immunization. This approach could inform the design and evaluation of next-generation vaccines that elicit strong protective immunity even in poor- or non-responder populations.

The use of a three-dose immunization regimen was based on preliminary findings showing that two doses of DNA or protein vaccines resulted in suboptimal or inconsistent antibody titers, particularly in BALB/c mice. The third dose was therefore introduced to enhance the magnitude and durability of humoral immunity, and to uncover differences in immune recall capacity between vaccine platforms. Notably, DNA + E protein domain III and E∆TM protein groups demonstrated substantial increases in ZIKV-specific IgG titers following the third dose, especially in C57BL/6 mice. In contrast, antibody responses in the formalin-inactivated and live virus groups plateaued after the second dose, suggesting a possible ceiling effect or antigen saturation. These findings highlight that the format, persistence, and immunological context of antigen exposure critically shape both the magnitude and kinetics of vaccine-induced immunity. The differential boosting capacity observed across platforms further underscores the importance of optimizing dosing schedules and considering host genetic background during preclinical vaccine development.

Limitations of this study include the relatively short post-immunization observation period, which may not adequately reflect the longevity of neutralizing antibody responses. Cellular immunity, including T cell activation and memory formation, was not evaluated and may reveal important strain-specific differences. Moreover, while mouse models are useful for comparative analysis, their applicability to human immune responses is limited. Future research should incorporate extended follow-up, in-depth immunophenotyping, and validation in additional animal models to strengthen and expand upon these findings.

In conclusion, our results demonstrate that host genetic background plays a pivotal role in shaping ZIKV vaccine-induced humoral immunity, particularly in the induction of neutralizing antibodies. C57BL/6 mice consistently outperformed BALB/c mice across vaccine platforms, highlighting their utility as a high-responder model. Conversely, the limited responses in BALB/c mice underscore the importance of incorporating low-responder models to comprehensively evaluate vaccine performance across diverse immunological landscapes.
